# Correlation Model of Damage Class and Deformation for Reinforced Concrete Beams Damaged by Earthquakes

**DOI:** 10.3390/ma18194638

**Published:** 2025-10-09

**Authors:** Chunri Quan, Ho Choi, Kiwoong Jin

**Affiliations:** 1 Department of Architecture, Graduate School and Faculty of Engineering, Osaka Institute of Technology, Osaka 5358585, Japan; chunri.quan@oit.ac.jp; 2 Department of Architecture, Faculty of Science and Technology, Shizuoka Institute of Science and Technology, Fukuroi 4370032, Japan; 3 Department of Architectural Engineering, Hannam University, Daejeon 34430, Republic of Korea; jink@hnu.kr

**Keywords:** RC beam, damage, ductility factor, shear strength margin, flexural behavior, residual seismic capacity, energy absorption capacity

## Abstract

The objective of this study was to propose a correlation model of the damage class and deformation of reinforced concrete (RC) beams damaged by earthquakes with a focus on columns and walls. For this purpose, a series of full-scale RC beam specimens with different shear strength margins were tested under cyclic lateral loading to examine their deformation performance and damage states. Then, the damage class and seismic capacity reduction factor of RC beams were evaluated based on the test results. The results showed that the tendency of shear failure, such as shear crack pattern and shear deformation component, of specimens with small shear strength margins was more remarkable, and its maximum residual crack widths tended to be slightly larger and dominated by shear cracks. The results also indicated that the effect of the shear strength margin on the seismic capacity reduction factor which represents the residual seismic performance of RC beams was limited, whereas the specimen with a smaller shear strength margin exhibited lower ultimate deformation capacity. In addition, there was a difference in the boundary value of the lateral drift angle which classifies the damage class of specimens with different shear strength margins. Finally, correlation models between the damage class and deformation of RC beams with different deformation capacities were proposed.

## 1. Introduction

The major concern of damaged reinforced concrete (RC) buildings after an earthquake is their safety regarding aftershocks, and quick damage inspections are needed. In the stage following quick damage inspections, a precise damage evaluation should be quantitatively performed to identify necessary actions required for the damaged buildings. For this purpose, the guidelines for Post-Earthquake Damage Evaluation and Rehabilitation (JBDPA, 2015) was developed in 1991 and revised in 2001 and 2015 in Japan [1]. In these guidelines, a residual seismic capacity ratio index, which corresponds to building damage, is defined as the ratio of capacity of the post-damaged condition to that of the pre-damaged condition (i.e., the ratio of the residual capacity to the original). Here, the original or residual capacity of RC buildings can be calculated based on the initial (pre-earthquake) or residual energy absorption capacity as defined in the Japanese Standard for Seismic Evaluation (JBDPA, 2017), which is most widely applied to evaluate the seismic capacity of existing pre-damaged buildings in Japan [2].

**Figure 1:**
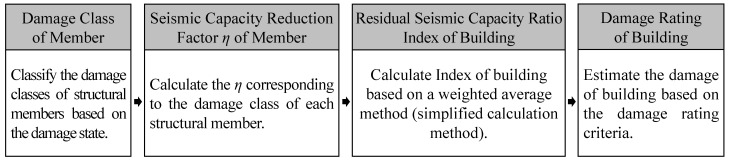
Basic concept of residual seismic capacity evaluation method.

Since the residual or dissipated energy of RC structures is difficult to grasp on-site, the current Japanese guidelines mentioned above also provide a simplified calculation method for the residual seismic capacity ratio index, which employs visual damage information such as the maximum residual crack widths of members. As shown in Figure 1, the damage classes of structural members should be classified first from the damage state [3,4]. Then, a seismic capacity reduction factor *η*, which is defined as the ratio of the absorbable hysteretic energy after an earthquake to the original absorbable energy of each structural member, should be calculated corresponding to the damage classes of members [5,6,7]. Considering the seismic capacity reduction factor *η*, a residual seismic capacity ratio index, which is defined as the ratio of post-earthquake seismic capacity to original capacity, can be calculated [8,9,10,11]. Finally, the damage of a building can be rated based on the damage rating criteria [12,13,14,15].

Jung et al. [16] conducted several earthquake response analysis studies for the evaluation of residual seismic capacity using the simplified calculation method described above. In this study, a correlation model between the damage class and deformation was assumed for the vertical members, such as columns and walls. Thereby, the damage classes of RC columns and walls could be estimated based on their deformation obtained from the analysis results. However, the validity of the correlation model between damage class and deformation for RC beam members, which exhibited significantly different deformation characteristics compared to the vertical members, has not yet been clearly verified. Therefore, it is difficult to evaluate the residual seismic capacity of weak-beam RC buildings through earthquake response analysis studies.

Therefore, in this study, static loading tests are conducted on two full-scale RC beam specimens with different shear strength margins to investigate the effects of the shear strength margin on the deformation capacity and damage pattern. Then, the damage classes for each specimen are discussed in detail based on the test results. Finally, we propose the correlation model between the damage class and ductility ratio which takes into account the deformation performance of RC beams.

## 2. Experiment Outline

### 2.1. Specimen Outline

**Figure 2:**
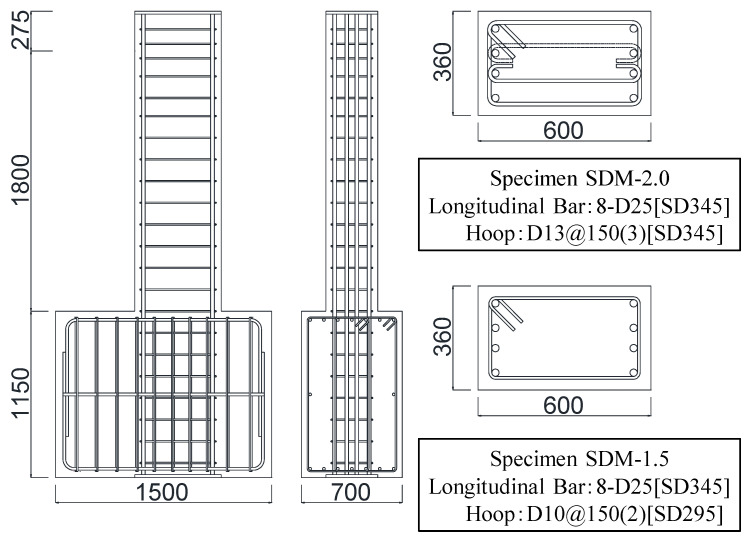
Details of the test specimens (unit: mm).

Figure 2, Table 1, Table 2, and Table 3 present the structural details, material characteristics, and specimen properties, respectively. Since the dimension effect on damage such as the residual crack width remains difficult to clarify, all specimens were designed as full-scale models to eliminate the influence of dimension effect. Then, the details of specimens were determined by referring to actual design examples, assuming beam members with a span length of 3600 mm [17]. As shown in Figure 2, each specimen had cross-sectional dimensions of 360 × 600 mm, with a length of 1800 mm to represent the geometric center of the actual span. The longitudinal reinforcement was 8-D25 (SD345, the standard yield strength of reinforcement was 345 MPa) and the spacing of transverse reinforcement was set at 150 mm for all specimens.

The calculated results of the ultimate flexural strength (*Q_mu_*) and the shear strength (*Q_su_*) using Equations (1) and (2) are summarized in Table 3. Here, the shear strength margin was defined as the ratio of *Q_su_* to *Q_mu_*, and *Q_su_* was adjusted using the reinforcement ratio and yield strength of transverse reinforcement, while *Q_mu_* was the same for each specimen [18]. Then, two specimens were fabricated with shear strength margins of 2.02 (specimen SDM-2.0; the transverse reinforcement ratio was 0.706% and the standard yield strength of transverse reinforcement was 345 MPa) and 1.57 (specimen SDM-1.5; the transverse reinforcement ratio was 0.264% and the standard yield strength of transverse reinforcement was 295 MPa).

(1) $$Q_{mu}=0.9a_{t}\sigma_{y}d/h_{w}$$

(2) $$Q_{su}=\left(\frac{0.068{p_{t}}^{0.23}\left(F_{c}+18\right)}{M/\left(Qd\right)+0.12}+0.85\sqrt{p_{w}\sigma_{wy}}\right)bj$$

where *a_t_* is the cross-sectional area of longitudinal tensile reinforcement (mm^2^), *σ_y_* is the yield strength of longitudinal reinforcement (MPa), *d* is the effective beam cross-section height (mm), *h_w_* is the beam length, *p_t_* is the longitudinal tensile reinforcement ratio (%), *F_c_* is the compressive strength of concrete (MPa), *M*/(*Qd*) is the shear span ratio, *p_w_* is the transverse reinforcement ratio, *σ_wy_* is the yield strength of transverse reinforcement (MPa), *b* is the beam cross-section width (mm), and *j* is the moment arm length from compressive to tensile resultant forces calculated by 7/8*d* (mm).

### 2.2. Loading Program and Instrumentation

**Figure 3:**
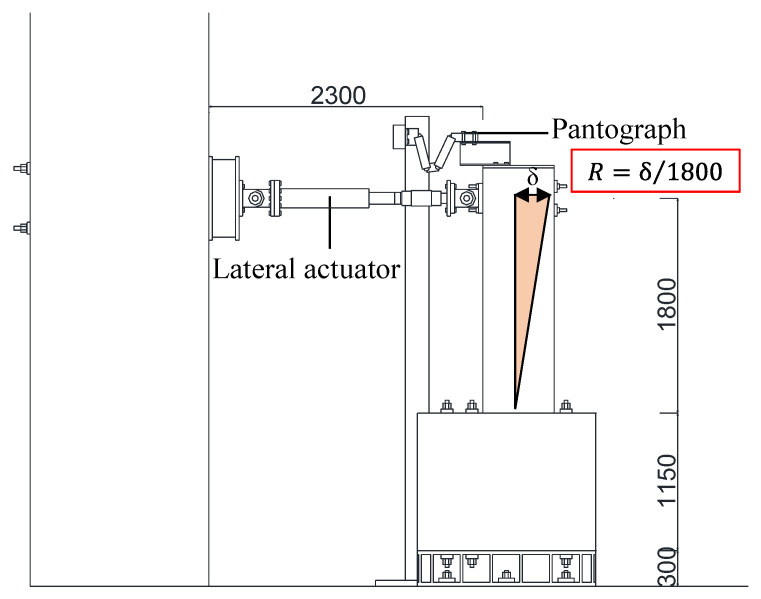
Test setup and loading configuration.

Figure 3 illustrates the loading system. The lateral actuator was positioned at the height of the specimen (at a beam length of 1800 mm) to produce a cantilever moment distribution, and a pantograph was installed above the specimen. The lateral load was applied under displacement control in both positive and negative directions, with one cycle for the lateral drift angles of 0.0625%, 0.125%, and 0.25% and two cycles for the lateral drift angles of 0.5%, 0.75%, 1.0%, 1.5%, 2.0%, 3.0%, 4.0%, and 5.0%. In this study, the lateral drift angle *R* is defined as the ratio of lateral displacement to the beam length of 1800 mm.

**Figure 4:**
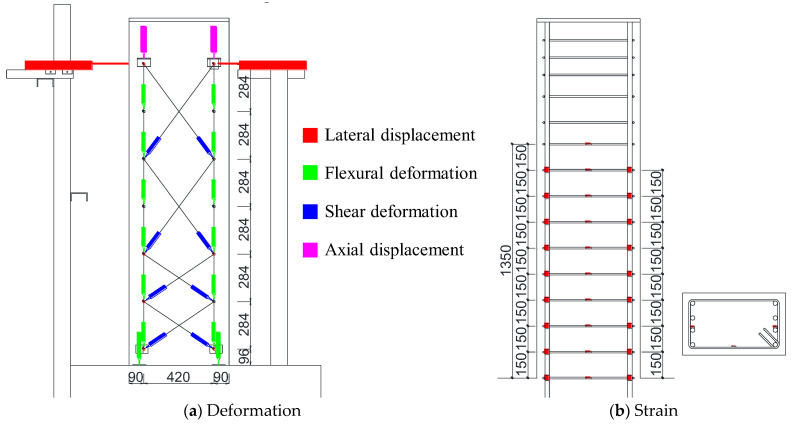
Measurement schemes.

As shown in Figure 4, flexural and shear deformations at each beam section were measured using displacement transducers (LVDTs). Strains in the longitudinal and transverse reinforcement were also recorded using strain gauges. Furthermore, the crack width and length were measured at the peak and unloading moment of each loading cycle for all lateral drift angles. Here, the crack width was measured visually using a crack scale, with the most open position value for each crack being recorded as its width. On the other hand, the crack length was calculated automatically using CAD software (AutoCAD 2020), which was digitized at the same scale after being measured visually. Detailed discussions on the flexural and shear deformations, as well as the damage, are presented in Section 3 and Section 4.

## 3. Experimental Results

### 3.1. Load–Deformation Relationship

**Figure 5:**
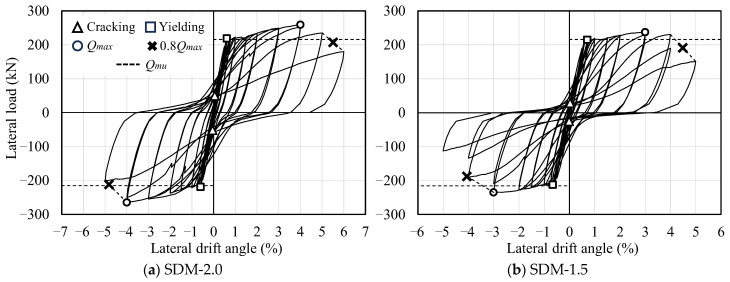
Hysteresis curves.

Figure 5 presents the load–deformation relationships of the two specimens, including the calculation results of the ultimate flexural strength (*Q_mu_*) explained earlier. In the figure, the symbols △, □, ○, and × indicate the points of first concrete crack observation, tensile longitudinal reinforcement yielding, maximum strength, and 80% of the maximum strength, respectively. The notable characteristics of the hysteresis curves observed in the experimental results are summarized as follows.

In specimen SDM-2.0, yielding of the tensile longitudinal reinforcement was observed at lateral drift angles of *R* = +0.61% and −0.60% under positive and negative loading, respectively. The maximum strengths of +259.5 kN and −265.0 kN were recorded at *R* = 4.0% of each loading direction, which were approximately 1.18 times higher than the calculated value (*Q_mu_*). The load bearing capacity remained above 80% of the maximum strength even at *R* = +5.50% and −4.83%.

Similar trends in the hysteresis response and maximum strength were also observed in specimen SDM-1.5. The yielding of the tensile longitudinal reinforcement was observed at lateral drift angles of *R* = +0.71% and −0.66% under positive and negative loading, respectively. The maximum strengths of +237.5 kN and −235.0 kN were recorded at *R* = 3.0% of each loading direction, which were approximately 1.07 times higher than the calculated value (*Q_mu_*). Thereafter, the load bearing capacity remained above 80% of the maximum strength even at *R* = +4.50% and −4.06%.

Based on the experimental results noted above, it was confirmed that the RC beam with a smaller shear strength margin had lower load bearing and deformation capacity, as shown in a previous study by Watanabe et al. [19].

### 3.2. Failure Pattern

**Figure 6:**
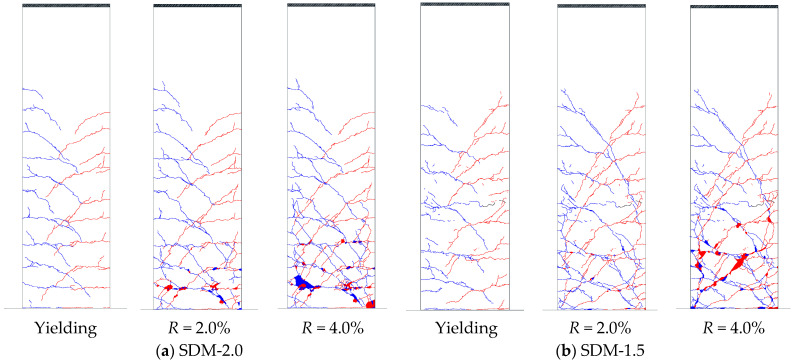
Damaged state (blue and red lines indicate cracks of positive and negative loading).

Figure 6 illustrates the crack patterns of each specimen at the loading stage of tensile longitudinal reinforcement yielding, as well as *R* = 2.0% and 4.0%. In the figure, the blue and red lines indicate cracks formed during positive and negative loading, respectively.

In specimen SDM-2.0, flexural cracks were observed along the critical section at a loading cycle of *R* = 0.0625%, and shear cracks occurred at a 0.5% loading cycle. After tensile longitudinal reinforcement yielding, crushing and spalling of the cover concrete occurred near the critical section at a 2.0% loading cycle. The damage, such as crack widths and concrete crushing area, extended intensely as the lateral drift angle increased; finally, crushing of the core concrete was observed with exposure of longitudinal reinforcement at a 5.0% loading cycle.

Compared to specimen SDM-2.0, SDM-1.5 showed similar crack and concrete spalling patterns until the 2.0% loading cycle. However, the tendency of the shear crack pattern and adhesion failure on the SDM-1.5 specimen with a smaller shear strength margin was more remarkable. In addition, crushing of the core concrete and exposure of longitudinal reinforcement appeared earlier at *R* = 4.0% in SDM-1.5.

### 3.3. Distribution of Shear Deformation

**Figure 7:**
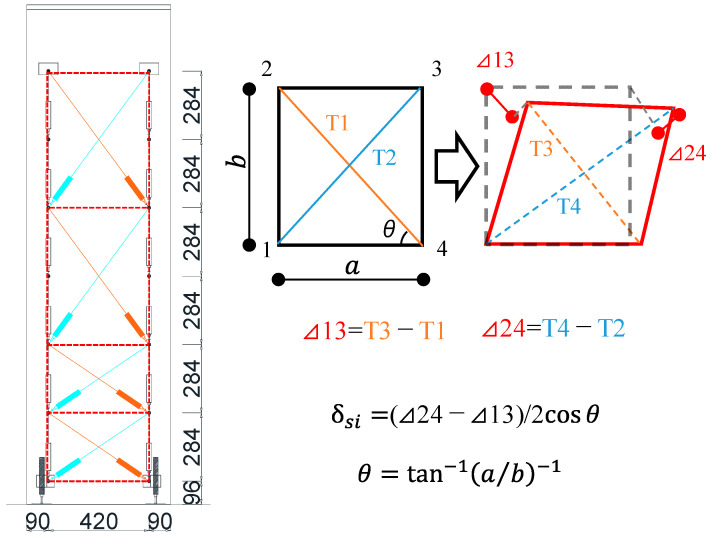
Calculation method for shear deformation.

**Figure 8:**
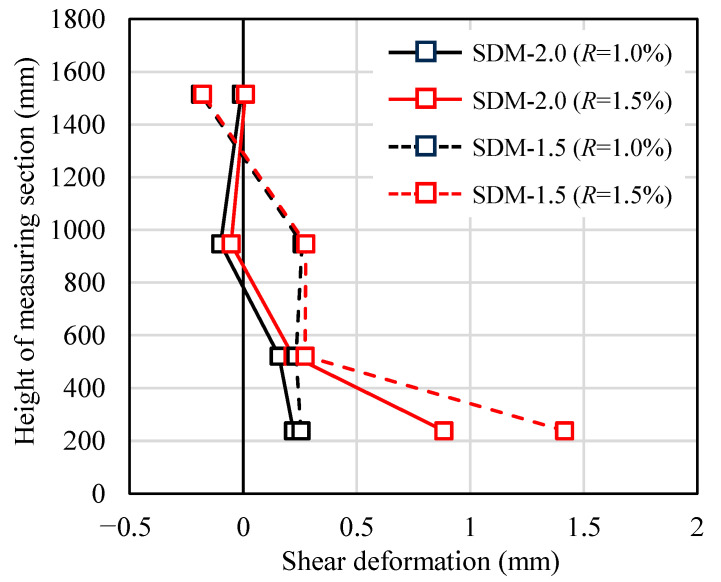
Comparison of shear deformation distribution.

Based on the previous research by Uchino et al. [20], as shown in Figure 7, the shear deformation of each measured section (*δ_si_*) in the axial direction of the specimens was calculated. Figure 8 illustrates the shear deformation distributions of both specimens at the drift angles of *R* = +1.0% and +1.5%. At *R* = +1.0%, the shear deformation at a height of approximately 200 mm above the critical section was close to each specimen. However, at *R* = +1.5%, the shear deformation at the same measured section was 1.42 mm for specimen SDM-1.5, which is approximately 1.6 times larger than that of specimen SDM-2.0. This indicates that the shear deformation of specimen SDM-1.5 tended to be greater than specimen SDM-2.0 at the subsequent drift angles, which means the shear deformation is more dominant in the RC beams with low shear strength margins, consistent with previous research conducted by Maeda et al. [21].

## 4. Evaluation of Damage Class and Seismic Capacity Reduction Factor *η*

### 4.1. Definition for Damage Class

**Figure 9:**
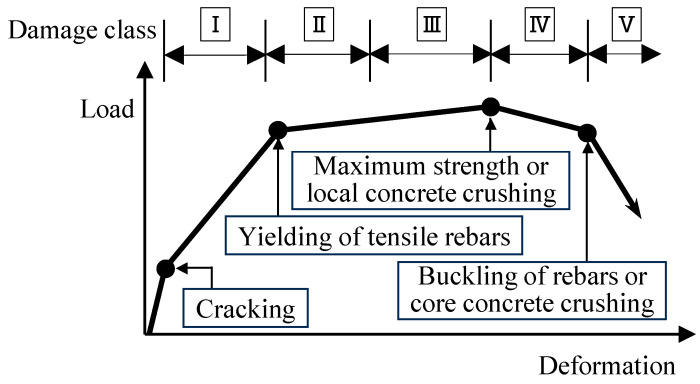
Damage class definition.

Damage classification of the specimens was performed based on the damage definition shown in Figure 9 and Table 4. The damage classes of the RC beams are originally defined based on the mechanical properties (engineering demand parameters), such as cracking of concrete, tensile longitudinal reinforcement yielding, and maximum strength in Japanese guidelines (JBDPA, 2015) [1]. Furthermore, the relationships between the damage class and visual damage information, such as maximum residual crack width, are also defined to easily classify the damage class on-site. Figure 9 schematically illustrates the load carrying capacity, load–deflection curve, and member damage class, and RC members are classified in one of five categories (I through V), as defined in Table 4.

### 4.2. Damage Class

**Figure 10:**
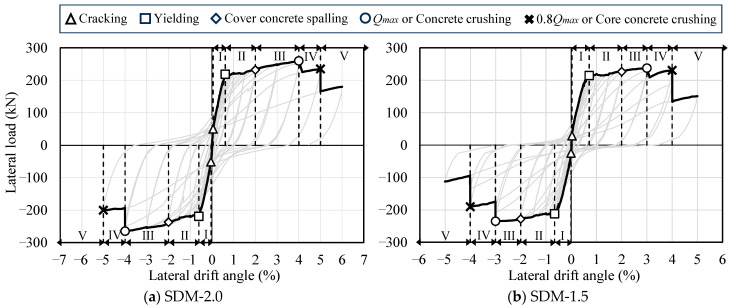
Damage class based on engineering demand parameters.

**Figure 11:**
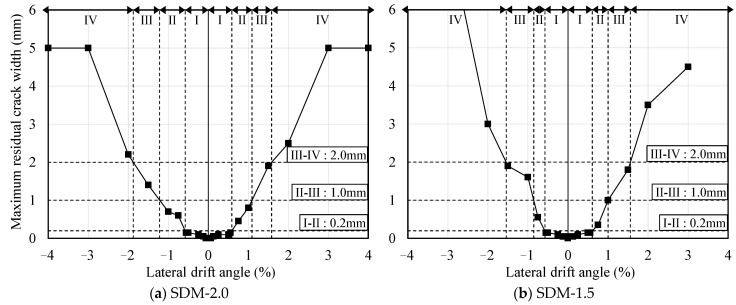
Damage class based on maximum residual crack widths.

Figure 10 and Figure 11 present the results of damage classification for each specimen based on the mechanical properties and maximum residual crack widths, respectively. In Figure 10, the symbols △, □, ◇, ○, and × indicate the points of concrete cracking, tensile longitudinal reinforcement yielding, local concrete crushing, maximum strength (or remarkable concrete crushing), and 80% of the maximum strength (or core concrete crushing), respectively. In both specimens, as shown in Figure 10 and Figure 11, the damage classes evaluated based on the maximum residual crack widths tended to be overestimated in the post-yield deformation region compared to those evaluated based on mechanical properties. This is due to the fact that the maximum residual crack widths were measured from limited cracks, which were observed in and near the critical section of the specimens.

Based on the damage class investigated above, the damage classes for each specimen were finally determined by comprehensively considering mechanical properties and damage development. Here, damage classes up to II were conservatively evaluated by comparing the damage state, including mechanical property variation and the maximum residual crack width, and the higher value was judged as the final damage class. On the other hand, damage classes III and IV were classified based on mechanical property variation, such as spalling of concrete and deterioration of bearing capacity, considering the influence of limited cracking on the maximum residual crack width for each specimen. In addition, a conservative evaluation was conducted for damage class V based on 80% reduction in maximum bearing capacity and core concrete crushing, which are widely used to define the safety limitation for RC structural members, and the higher value was selected.

Table 5 shows the final damage class evaluation results and boundary values of the lateral drift angle to classify the damage class for each specimen. The boundary values of the lateral drift angle that classify damage classes up to III were similar for both specimens. However, as discussed above, because the shear strength margin influenced the deformation capacity and shear deformation distribution of RC beams, the boundary values of the lateral drift angle that classified damage classes above III tended to be smaller in specimen SDM-1.5 compared to SDM-2.0.

### 4.3. Seismic Capacity Reduction Factor η

**Figure 12:**
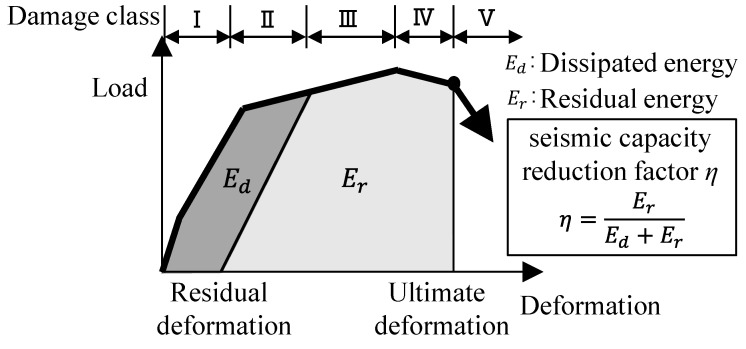
Conceptual diagram of seismic capacity reduction factor *η*.

**Figure 13:**
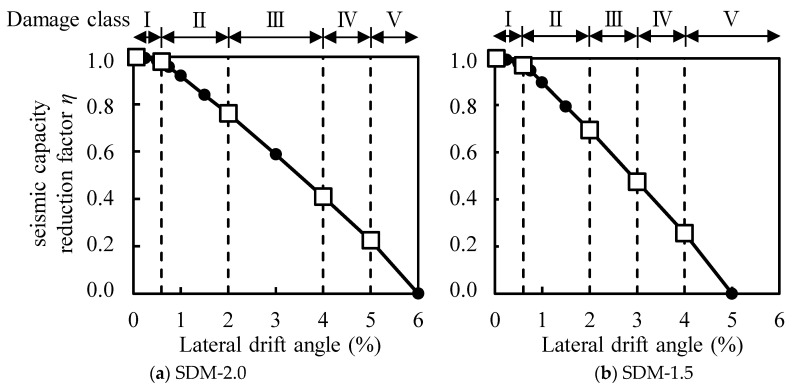
Calculated results of factor *η*.

The seismic capacity reduction factor *η* of each specimen was calculated based on the previous method developed in the Japanese guidelines (JBDPA, 2015) [1], which is defined as the ratio of the absorbable hysteretic energy after an earthquake to the original absorbable energy of structural members, as illustrated in Figure 12. Figure 13 shows the calculated result of factor *η* for each specimen relating to the damage classes determined in Section 4.2. Table 6 arranges the boundary value of factor *η* to classify the damage class for each specimen, as shown in Figure 13 with the symbol □, comparing it to the values of the ductile beam defined in the Japanese guidelines (JBDPA, 2015) [1]. Here, factor *η* was calculated using a positive loading test only. It was found that the effect of the difference in shear strength margins on the seismic capacity reduction factor *η* was limited, and the boundary value of factor *η* was generally consistent with the specified value for the ductile beam.

## 5. Correlation Model of Damage Class and Deformation

The ductility ratio *μ* was applied to develop the correlation model between damage class and deformation. Here, *μ* is defined as the ratio of the empirical displacement to the yielding displacement. As mentioned above, yielding displacement was confirmed by the strain gauges installed to the longitudinal reinforcement of each specimen. Table 7 shows the boundary values of the ductility ratio that classify the damage classes. Comparing these values of both specimens, the trend was generally similar up to damage class III. Meanwhile, from damage class III onward, a difference in the boundary values of the ductility ratio was observed depending on the shear strength margin of the specimen.

**Figure 14:**
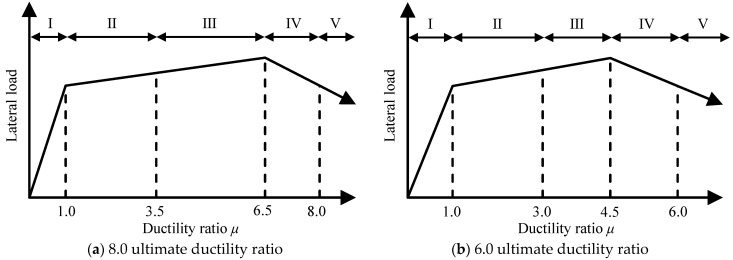
Correlation model of damage class and ductility ratio *μ*.

Figure 14 shows the proposed correlation models between damage class and ductility ratio for flexural RC beams with ultimate ductility ratios of 6 and 8. Here, the boundary values of the ductility ratio were simplified by applying the approximate values of the detailed calculation results, as shown in Table 8, and the detailed calculation results adopted smaller values for positive and negative loading, as discussed in Table 7. As a result, the correlation model was established by using the ultimate ductility ratio to represent the difference in deformation capacity of RC beams, and the boundary values of the ductility ratio were proposed for classifying damage classes; these would also be applicable to estimate the damage classes of RC beams based on their deformation.

## 6. Conclusions

This paper presented the results of static loading tests on RC beam specimens with different shear strength margins, showing the effect on the deformation capacity and damage pattern of RC beams. Then, the relationship between the damage and deformation of RC beams was investigated. The key findings of this study are summarized as follows:

1. Based on the test results, the RC beam specimen with a smaller shear strength margin showed lower lateral load bearing capacity and deformation performance. In this specimen, the failure pattern was also different, for example, the tendency of shear failure was more remarkable, and shear deformation was more dominant.
2. The damage classification of RC beam specimens was successfully performed based on the realignment concept, which comprehensively considers mechanical properties and damage development, such as the cracking and crushing of concrete, yielding of reinforcement, and deterioration of lateral load bearing capacity.
3. The effect of the shear strength margin on the seismic capacity reduction factor *η* of RC beams was limited, whereas the specimen with a smaller shear strength margin exhibited lower ultimate deformation capacity.
4. The boundary values of the seismic capacity reduction factor *η* to classify damage classes were similar for both specimens, and they were generally consistent with the specified values for ductile beams in the Japanese guidelines.
5. The boundary values of the ductility ratio *μ* to classify damage classes in both specimens generally showed similar trends up to damage class III. However, after experiencing damage class III, the boundary values of the ductility ratio *μ* were lower in the specimen with a smaller shear strength margin.
6. Correlation models of damage class and ductility ratio were proposed by applying ultimate ductility ratios of 6 and 8 to represent the difference in deformation capacity of RC beams, and the boundary values of the ductility ratio *μ* for classifying damage classes were also specified.

This paper focused on the relationship between damage class and deformation of RC beams. The proposed correlation models of damage class and deformation should be applicable to future analytical studies focused on evaluating the residual seismic capacity of weak-beam RC structures. Moreover, since this study utilized two specimens with different shear strength margins adjusted using the transverse reinforcement ratio and yield strength of transverse reinforcement, loading tests should be performed on specimens with varying deformation capacities, and their influencing factors should be assessed to further enhance the applicability of the correlation models.

## Figures and Tables

**Table 1:** Mechanical properties of concrete (unit: MPa).

Specimen	Elastic Modulus	Compressive Strength	Tensile Strength
Specimen SDM-2.0	2.67 × 10^4^	29.6	2.75
Specimen SDM-1.5	2.66 × 10^4^	31.4	3.06

**Table 2:** Mechanical properties of reinforcement materials (Unit: MPa).

Rebar Specification	Elastic Modulus	Yield Strength	Tensile Strength
D25 (SD345)	1.99 × 10^5^	394.4	576.4
D13 (SD345)	1.95 × 10^5^	399.3	554.6
D10 (SD295) *^a^	1.78 × 10^5^	364.2	486.7

*^a^ Yield strength obtained by 0.2% offset method.

**Table 3:** Summary of specimen properties.

Specimen	SDM-2.0	SDM-1.5
Cross-section	360 mm × 600 mm	
Length	1800 mm	
Longitudinal reinforcement	8-D25 [SD345]	
Longitudinal tensile reinforcement ratio	1.04%	
Transverse reinforcement	D13@150(3) [SD345]	D10@150(2) [SD295]
Transverse reinforcement ratio	0.706%	0.264%
Ultimate flexural strength *Q_mu_*	218.6 kN	
Shear strength *Q_su_*	441.5 kN	343.2 kN
*Q_su_*/*Q_mu_*	2.02	1.57

**Table 4:** Damage class criteria for ductile RC members [1].

Damage Class	Description of Damage
I	Visible narrow cracks on concrete surface (Crack width is less than 0.2 mm)
II	Visible clear cracks on concrete surface (Crack width is about 0.2–1.0 mm)
III	Local crush of concrete cover, remarkable wide cracks (Crack width is about 1.0–2.0 mm)
IV	Remarkable crush of concrete with exposed reinforcing bars, spalling of concrete cover (Crack width is more than 2.0 mm)
V	Buckling of reinforcing bars, cracks in core concrete, visible vertical and/or lateral deformation in columns and/or walls, visible settlement and/or leaning of the building

**Table 5:** Boundary value of lateral drift angle *R* for damage class.

Damage Class	SDM-2.0		SDM-1.5	
	Positive Loading	Negative Loading	Positive Loading	Negative Loading
0				
	+0.06%	−0.05%	+0.03%	−0.01%
I				
	+0.59%	−0.58%	+0.60%	−0.58%
II				
	+2.0%	−2.0%	+2.0%	−2.0%
III				
	+4.0%	−4.0%	+3.0%	−3.0%
IV				
	+5.0%	−4.83%	+4.0%	−4.0%
V				
	

**Table 6:** Relationship between damage class and factor *η*.

Damage Class	SDM-2.0	SDM-1.5	Ductile Beam *^b^
I	0.98	0.97	0.95
II	0.76	0.70	0.75
III	0.43	0.48	0.5
IV	0.22	0.25	0.2
V	0	0	0

*^b^ Defined values in the guidelines (JBDPA, 2015) [1].

**Table 7:** Boundary value of ductility ratio *μ* for damage class.

Damage Class		0–I	I–II	II–III	III–IV	IV–V
SDM-2.0	Positive loading	0.09	0.96	3.27	6.55	8.19
	Negative loading	0.08	0.96	3.34	6.68	8.06
SDM-1.5	Positive loading	0.04	0.85	2.82	4.24	5.65
	Negative loading	0.01	0.88	3.03	4.55	6.07

**Table 8:** Proposal of relationship between damage class and ductility ratio *μ*.

Damage Class		I–II	II–III	III–IV	IV–V	*μ_u_* *^c^
SDM-2.0	Detailed	0.96	3.27	6.55	8.06	8.06
	Simplified	1.0	3.5	6.5	8.0	8.0
SDM-1.5	Detailed	0.85	2.82	4.24	5.65	5.65
	Simplified	1.0	3.0	4.5	6.0	6.0

*^c^ Ultimate ductility ratio.

## Data Availability

The original contributions presented in this study are included in the article. Further inquiries can be directed to the author.

## References

[1] The Japan Building Disaster Prevention Association (JBDPA) (1991). Guidelines for Post-Earthquake Damage Evaluation and Rehabilitation.

[2] The Japan Building Disaster Prevention Association (JBDPA) (1977). Standard for Seismic Evaluation of Existing Reinforced Concrete Buildings.

[3] Bunno M., Maeda M., Nagata M. (2000). A study of the damage level estimation of RC buildings based on residual seismic capacity of members. Proc. Jpn. Concr. Inst..

[4] Maeda M., Bunno M. Post-earthquake damage evaluation for RC buildings based on residual seismic capacity in structural members. Proceedings of the US-Japan Workshop on Performance Based Seismic Design of Reinforced Concrete Building Structures.

[5] Bunno M., Yukimura N., Maeda M., Kabeyazawa T. (1999). Experimental study on behavior of reinforced concrete beams under axial restriction. Proc. Jpn. Concr. Inst..

[6] Bunno M., Nagayama K., Maeda M. (2001). An evaluation of residual seismic capacity of reinforced concrete columns based on structural damage. Proc. Jpn. Concr. Inst..

[7] Ito S., Takahashi K., Maeda M. (2013). Evaluation on residual seismic capacity of reinforced concrete columns and walls with shear failure. Proc. Jpn. Concr. Inst..

[8] Bao S., Matsukawa K., Maeda M. Residual seismic capacity evaluation method for RC buildings with weak beam. Proceedings of the 13th Japan Earthquake Engineering Symposium.

[9] Miura K., Maeda M., Matsukawa K., Takahashi K. (2012). Evaluation method of contribution factor of structural member for seismic capacity of single-story RC frames considering strength and energy dissipation (part 1). J. Struct. Constr. Eng..

[10] Quan C., Takahashi N., Choi H., Nakano Y. (2013). Residual seismic capacity evaluation of overall weak-beam RC frame based on energy absorption capacity. J. Struct. Constr. Eng..

[11] Fujita K., Miura K., Tabata Y., Maeda M., Shegay A., Seki M. (2021). Post-earthquake capacity evaluation of RC frame structures with multi-story flexural walls. J. Struct. Constr. Eng..

[12] Architectural Institute of Japan (AIJ) (1968). Reconnaissance Report of the 1968 Tokachi-Oki Earthquake.

[13] Architectural Institute of Japan (AIJ) (1978). Reconnaissance Report of the 1978 Miyagiken-Oki Earthquake.

[14] Architectural Institute of Japan (AIJ) (1995). School Buildings Reconnaissance Report of the 1995 Kobe Earthquake.

[15] Institute of Industrial Science of the University of Tokyo (IIS) (2012). Earthquake and Tsunami Reconnaissance Report of the 2011 Great East Japan Earthquake.

[16] Jung M., Maeda M., Tasai A., Nagata M. (2002). Estimation of residual seismic performance for RC buildings damaged due to earthquake. J. Struct. Eng..

[17] Architectural Institute of Japan (AIJ) (1965). Structural Design of Reinforced Concrete Buildings.

[18] Architectural Institute of Japan (AIJ) (1971). AIJ Standard for Structural Calculation of Reinforced Concrete Structures.

[19] Watanabe H., Korenaga T., Nakano K., Matsuzaki Y. (2002). Experimental study on evaluation of ductility of reinforced concrete beams. J. Struct. Constr. Eng..

[20] Uchino S., Sato R., Tajima K., Shirai N. (2015). Effectiveness of steel brace reinforcement for a row-span RC frame with walls using round steel bars as main reinforcement. Proc. Jpn. Concr. Inst..

[21] Maeda M., Arizono Y., Yukimura N. (1997). Experimental Study on Evaluation of Deformation in R/C Beams. Proc. Jpn. Concr. Inst..

